# Oxidative Stress in Hypertensive Patients Induces an Increased Contractility in Vein Grafts Independent of Endothelial Function

**DOI:** 10.4061/2011/902129

**Published:** 2011-11-13

**Authors:** Claudio Joo Turoni, Rodrigo Marañón, Maria Karbiner, Juan Muntaner, Víctor Proto, María Peral de Bruno

**Affiliations:** ^1^Departamento de Fisiología, Facultad de Medicina Universidad Nacional de Tucumán INSIBIO-CONICET, Tucumán, Argentina; ^2^Centro Modelo de Cardiología S.R.L., Balcarce 32, 4000 Tucumán, Argentina

## Abstract

*Objective*. To evaluate the impact of oxidative stress on vascular reactivity to vasoconstrictors and on nitric oxide (NO) bioavailability in saphenous vein (SV) graft with endothelial dysfunction from hypertensive patients (HT). *Methods*. Endothelial function, vascular reactivity, oxidative state, nitrites and NO release were studied in isolated SV rings from HT and normotensive patients (NT). Only rings with endothelial dysfunction were used. *Results*. HT rings presented a hyperreactivity to vasoconstrictors that was reverted by diphenylene iodonium (DPI). In NT, no effect of DPI was obtained, but N*ω*-nitro-_L_-arginine methyl ester (L-NAME) increased the contractile response. NO was present in SV rings without endothelial function. Nitrites were higher in NT than in HT (1066.1 ± 86.3 pmol/mg; *n* = 11 versus 487.8 ± 51.6; *n* = 23; *P* < 0.01) and inhibited by nNOS inhibitor. L-arginine reversed this effect. Antioxidant agents increased nitrites and NO contents only in HT. The anti-nNOS-stained area by immunohistochemistry was higher in NT than HT. HT showed an elevation of oxidative state. *Conclusions*. Extraendothelial NO counter-regulates contractility in SV. However, this action could be altered in hypertensive situations by an increased oxidative stress or a decreased ability of nNOS to produce NO. Further studies should be performed to evaluate the implication of these results in graft patency rates.

## 1. Introduction

In coronary artery bypass grafting surgery (CABGS), the vessels that must be used are internal mammary arteries (IMA) and saphenous veins (SV); however, SVs present a greater risk of occlusion [1]. Nitric oxide (NO) could be involved in the improvement of SV graft patency rates [2, 3] since NO plays a pivotal role in vascular homeostasis [4–6]. Three isoforms of NO synthase (NOS) exist: neural (nNOS), inducible (iNOS), and endothelial (eNOS) [7]. Although nNOS was first described in neurons, it is also present in vascular smooth muscle cell (VSMC) [8]. We have shown in IMA that extraendothelial NO is released from nNOS present in VSMC [9, 10]. 

Oxidative stress plays a role in NO bioavailability [11]. Some studies have reported that superoxide (O_2_^−^) contributes to the development of hypertension [12, 13]. In experimental hypertension, Li et al. [14] have found elevated O_2_^−^ levels in veins, suggesting that NADPH oxidase activity induces hyperreactivity to vasoconstrictors. In agreement with these data, we have observed that enhanced NADPH oxidase activity drives O_2_^−^ production in genetically hypertensive rats [15]. In human IMA, we found that extraendothelial NO counter-regulates angiotensin II (Ang II) contractility and that this action is altered in hypertension, probably by an increased oxidative stress and a decreased nNOS ability to produce NO [9, 10]. In addition, in human umbilical endothelial cells, NADH-/NADPH-oxidases play a role in O_2_^−^ induced by Ang II [16]. In SV, antioxidant agents improve endothelial function [17].

Alterations of the vascular reactivity, NO bioavailability, and oxidative stress could affect SV patency rates. This notion would be supported by some reports that indicated that functional and structural abnormalities of SV result in graft thrombosis, intimal hyperplasia, and occlusion [3, 18]. 

To the best of our knowledge, despite the fact that hypertension is a major risk factor for coronary disease, the impact of hypertension and the role of oxidative stress in the contractile function of SV grafts have not been studied.

The objective of this work was to evaluate the impact of oxidative stress on Ang II and norepinephrine-(NE-) mediated vascular reactivity and NO bioavailability in SV grafts of hypertensive patients (HT).

## 2. Materials and Methods

Discarded SV segments were obtained from CABGS (Centro Modelo de Cardiología, Tucumán, Argentina). To establish the impact of hypertension on oxidative stress, NO contents and vascular contractility and strict inclusion criteria in relation to the risk factors were taken into account. Patients with diabetes, renal failure, pulmonary disease, peripheral vascular disease in a clinical report, uncontrolled dyslipemia, or active smoking at the time of surgery were not included. To test the influence of hypertension, patients were divided into two groups, hypertensive (HT) and normotensive (NT), according to clinical report. The clinical characteristics are shown in Table 1. Blood pressure was controlled at the time of CABGS. Informed consent according to institutional guidelines was obtained from each patient.

### 2.1. Vessel Preparation

After surgery, SVs were immediately placed in Krebs solution (mM: NaCl 118.3; KCl 4.7; CaCl_2_ 2.5; MgSO_4_ 1.2; KH_2_PO_4_ 1.2; NaHCO_3_ 25; Glucose 11.1; Na_2_EDTA 0.026), maintained at 4°C, and transferred to the laboratory. Each vessel was dissected free from connective tissue, and 2 to 5 rings (5 mm) were obtained.

### 2.2. Isometric Tension Measurement

Rings were mounted between two stainless-steel wires in organ chambers filled with Krebs solution, which had been gassed with 95% O_2_ and 5% CO_2_ (pH 7.4). One wire was anchored, and the other one was connected to an isometric force transducer (Gould UC2, USA) and a recorder (Kipp and Zonen BD41, Holland). Isometric tension was measured under an initial tension of 3 g, which was found to be the optimal tension in which the depolarizing high K^+^ solution induced contraction [19]. All preparations were equilibrated and washed every 15 minutes during 120 minutes. 

To evaluate endothelial function, a cumulative dose response curve (CDRC) to acetylcholine (Ach) (10^−8^–10^−4^ M) in precontracted NE (10^−6^ M) rings was performed. Endothelial function was considered to be present when rings relaxed in response to Ach. Similar Ach responses were observed in all rings from the same patient, which is necessary to point out. The absence of endothelium-dependent relaxation was observed in rings from 86.4% of the patients (NT: 12; HT: 26 patients). According to the objectives of the present work, only these rings (*n* = 38) were used. In this regard, to rule out the presence of endothelium, immunohistochemical studies were performed (see below). To test endothelium-independent relaxation, sodium nitroprusside (SNP) 10^−5^ M was added to NE-precontracted rings. In all cases, SNP induced a nearly complete relaxation (HT: 99 ± 6% and NT: 107 ± 11% of NE pre-contraction; *P*: NS).

### 2.3. Reactivity to Ang II and NE

Ang II CDRC (10^−10^–10^−5^ M) or NE CDRC (10^−9^–10^−5^ M) were performed. The maximal contractile response (*R*_max⁡_) and negative log of the molar concentration inducing 50% of the *R*_max⁡_ (pEC_50_) were measured from the corresponding CDRC. 

To evaluate the effect of NO-mediated inhibition on Ang II and NE reactivity, rings were pretreated with N*ω*-nitro-_L_-arginine methyl ester (L-NAME: 10^−4^ M) for 30 min. 

To evaluate the possible role of oxidative stress in vascular reactivity, rings were pretreated with diphenylene iodonium (DPI: 10^−5^ M) or tempol (10^−4^ M) for 30 min.

At the end of the experiments, vascular reactivity was checked with 100 mM KCl. Data with respect to tension were presented in milligrams (mg).

### 2.4. Calculation of Nitrites

Nitrites were measured by the Griess reaction, which is frequently used to indirectly measure NO contents [20]. We have previously shown that stretching is an ideal condition for *in vitro* nitrite dosage in isolated human vessels [9, 10]. Therefore, simultaneous* in vitro* measurements of vascular reactivity and nitrite release were performed under 3 g of preload. The absorbance was measured spectrophotometrically at 540 nm. Data with respect to tissue were expressed in pmol/mg of tissue.

To corroborate an extraendothelial NO presence, some rings were rubbed. The possible NOS isoforms involved were evaluated by treatment with L-NAME 10^−4^ M, L-NAME plus L-arginine 10^−2^ M, S-methyl-_L_-thiocitrulline 10^−5^ M (nNOS inhibitor), or aminoguanidine 10^−4^ M (iNOS inhibitor). Because constitutive NOS has been described as a Ca^2+^/calmodulin-dependent enzyme, some rings were incubated in Ca^2+^-free media (Krebs solution without CaCl_2_ plus EGTA 3 mM). 

The role of oxidative stress on nitrite contents was evaluated with DPI 10^−5^ M or tempol 10^−4^ M.

### 2.5. Direct Measurement of NO

NO release was evaluated in real time, with the membrane-type NO-sensitive electrode (ISO-NOP; WPI, USA). This electrode records an electrical current that is directly proportional to the NO concentration [21]. The signal was acquired by the Apollo 4000 recording system (WPI, USA). After 120 minutes of equilibration, the electrode was stabilized and the baseline of the current became stable. Data were expressed in nanoAmpers (nA). 

The role of oxidative stress in the NO content was evaluated with tempol 10^−4^ M.

### 2.6. Histological Studies

At the end of the experiments, the rings were fixed in buffered formol and stained with hematoxylin and eosin (H&E). Immunohistochemistry examination of endothelium was performed with monoclonal CD34 antibodies (Clone: Qbend 10; Bio Genex, USA; dilution: 1/160) [22], anti-eNOS antibody (Santa Cruz, USA, dilution 1/100). In all cases, the absence of an endothelial cell layer was observed by H&E, anti-CD-34, and anti-eNOS staining.

nNOS examination was performed with rabbit anti-nNOS antibody (BD Bioscience Pharmingen; dilution: 1 : 100) [9]. The positive control for VSMC presence was performed with anti-*α* actin antibodies (Sigma Chemical Company, USA) [23].

The anti-nNOS antibody-stained area was calculated using a light microscope connected to a video camera and an informatics system (Image J 1.43 *μ*, MD, USA) calibrated to correspond to an equivalent *μ*m^2^ value (1 *μ*m^2^ = 18.400 px), and the anti-nNOS-stained area/total area ratio was calculated.

### 2.7. Determination of Oxidative State

To evaluate the oxidative state protein carbonyl content, conjugated diene (CD) (the primary product of lipid peroxidation), the reduced/oxidized glutathione (GSH/GSSG) ratio, and thiobarbituric acid (TBA) reactive substances (TBARS) were determined.

Rings were homogenized in ice-cold Tris-KCl buffer (0.15 M, pH 7.4), precipitated by trichloroacetic acid (TCA: 20%), and centrifuged. The pellet was incubated with 2,4-dinitrophenylhydrazine (DNPH: 0.002 g/mL), washed with an ethanol-ethylacetate mixture, centrifuged, and redissolved with guanidine HCl/dithiothreitol. Protein carbonyl contents were read spectrophotometrically at 370 nm [24] and expressed as pmol/mg protein.

CD was determined spectrophotometrically at 233 nm using a chloroform-methanol mixture [25] and was correlated with mg of phosphates. 

Glutathione was determined by a spectrophotometric method [26]. To calculate total glutathione, fractions were homogenized in phosphate buffer plus EDTA 6.3 mM (pH 7.4), mixed with TCA 10%, and centrifuged. The supernatant was incubated with NADPH 0.3 mM, DTNB 6 mM, and glutathione reductase 0.077 U (10 *μ*L). To calculate GSH, 20 *μ*L of the supernatant was incubated for 15 min with DTNB 6 mM (0.1 mL). GSSG was calculated by subtracting GSH from the total glutathione. Absorbance was read spectrophotometrically at 412 nm and correlated with protein contents, and then the GSSG/GSH ratio was calculated.

Lipid peroxidation was evaluated in SV fractions homogenized in detergent-free buffer by measurement of malondialdehyde contents [27]. The absorbance was read spectrophotometrically at 535 nm. The results were expressed as *μ*mol TBARS/mg of protein, as determined by the Lowry's method.

### 2.8. Data Analysis

Data were expressed as mean ± standard error (SE).

To calculate pEC_50_ for each CDRC, the sigmoid equation of the curve fitting program “Graph-Pad” Prism 3.0 was used. Paired or nonpaired Student's *t*-test and ANOVA with Newman-Keuls tests were used when appropriate. Results were considered to be significant when *P* < 0.05.

## 3. Results

### 3.1. Reactivity to Ang II and NE

**Figure 1:**
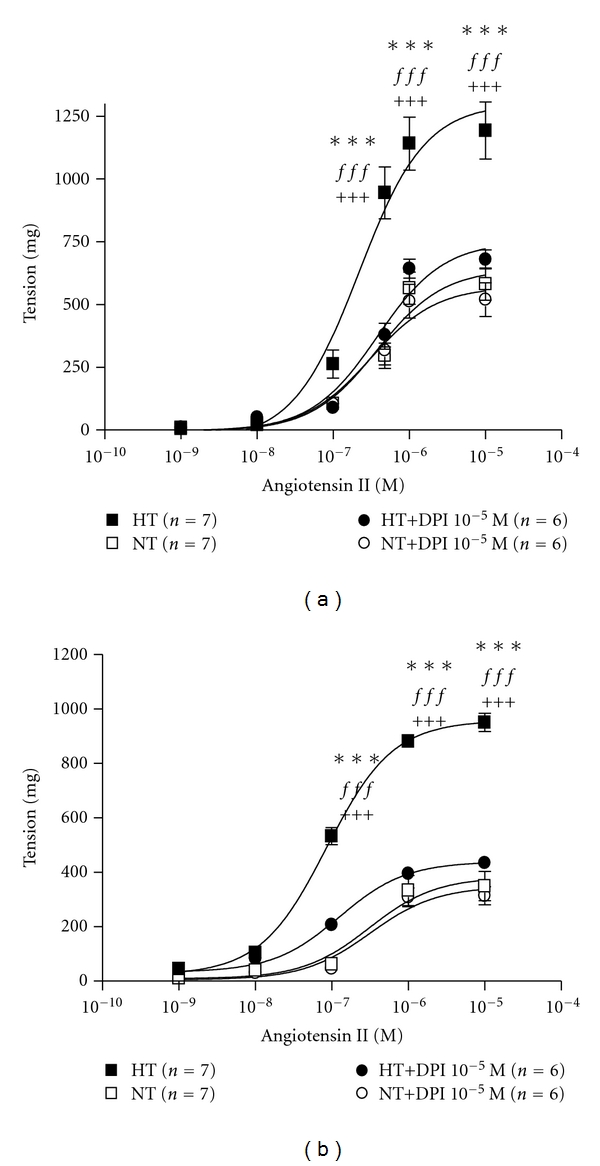
Cumulative dose-response curves (CDRC) to agonists in SV rings. (a) CDRC to Angiotensin II (Ang II) in HT (black) and NT (white) patients in the absence (squares) or presence (circles) of DPI. ***P < 0.001 HT versus NT. *ff*: *P* < 0.03 HT versus HT+DPI; *ff* *f*: *P* < 0.001 HT versus HT+DPI. ^+++^ *P* < 0.001 HT versus NT+DPI. (b) CDRC to norepinephrine (NE) in HT (black) and NT (white) patients in the absence (squares) or presence (circles) of DPI. ***P < 0.001 HT versus NT. *ff*: *P* < 0.03 HT versus HT plus DPI; *ff* *f*: *P* < 0.001 HT versus HT plus DPI. ^+++^ *P* < 0.001 HT versus NT plus DPI. Data are expressed as mean ± standard error. The number of rings is given in parentheses.

Figure 1 shows the vasocontractile response to Ang II (Figure 1(a)) and NE (Figure 1(b)) on SV rings without endothelium and the effect of DPI in this response. 

 Ang II (10^−9^–10^−5^ M) produced a dose-dependent contraction in both NT and HT rings (Figure 1(a)); however, HT showed higher Ang II *R*_max⁡_ values (Table 2). A curve fitness analysis showed no difference in potency (pEC_50_) between NT and HT (−6.5 ± 0.2; *n* = 7 versus −6.6 ± 0.1; *n* = 6, resp.). In HT, DPI decreased the Ang II dose-dependent contraction (Figure 1(a)) and *R*_max⁡_ (Table 2), however, this agent did not modify the Ang II response in NT. Tempol did not modify the Ang II *R*_max⁡_ in HT or NT (Table 2).

Similar to Ang II, NE (10^−9^–10^−5^ M) produced a dose-dependent contraction in both NT and HT (Figure 1(b)). The NE *R*_max⁡_ was higher in HT (Table 2). No difference in the NE pEC_50_ values between NT and HT was found (−7.6 ± 0.6; *n* = 6 versus −7.1 ± 0.1; *n* = 6). In HT, DPI decreased the NE dose-dependent contraction (Figure 1(a)) and *R*_max⁡_ (Table 2), however, this agent did not modify the NE response in NT. Tempol did not modify the NE *R*_max⁡_ in HT or NT (Table 2). 

In HT, L-NAME 10^−4^ M did not modify the Ang II *R*_max⁡_, however, it increased the Ang II reactivity in NT (Table 2). L-NAME did not modify the NE *R*_max⁡_ in HT, but it did increase the NE reactivity in NT (Table 2). Either Ang II or NE L-NAME modified the pEC_50_. 

In contrast to Ang II and NE, the response to KCl 100 mM was similar in HT (1115.9 ± 141.7 mg; *n* = 22) and NT (1056.7 ± 193.8 mg; *n* = 10).

### 3.2. NO Release

Nitrite contents were present in both NT and HT (1066.1 ± 86.3 pmol/mg; *n* = 11 versus 487.8 ± 51.6; *n* = 23; *P* < 0.01), despite the endothelial absence. Moreover, rubbed maneuvers did not blunt the nitrite contents in either NT (Δ: 5 ± 13%, *n* = 8; *P*: NS) or HT (Δ: 6 ± 12%, *n* = 10; *P*: NS).

**Figure 2:**
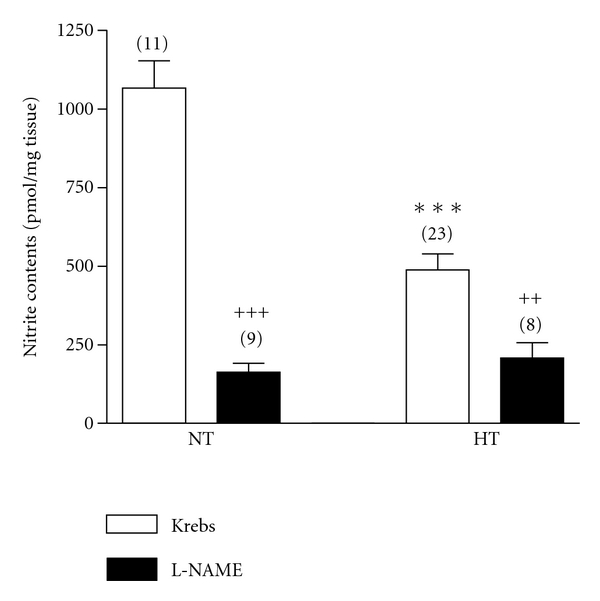
Effect of L-NAME on nitrite contents of NT and HT SV rings. White bars: Krebs. Black bars: L-NAME 10^−4^ M. ***P < 0.001 HT versus NT. ^++^ *P* < 0.01 HT versus HT plus L-NAME. ^+++^ *P* < 0.01 NT versus NT pus L-NAME. Data are expressed as mean ± standard error. The number of rings is given in parentheses.

Figure 2 shows the effect of L-NAME on nitrite contents. L-NAME significantly decreased nitrites in all cases. No differences between NT and HT in the presence of L-NAME were found (Figure 2, black bars). L-NAME plus L-arginine did not modify the nitrite contents in HT (Δ: 11.9 ± 4.2 pmol/mg; *n* = 10) and NT (Δ: 15.4 ± 5.2 pmol/mg; *n* = 6). 

Similarly to L-NAME, S-methyl-L-thiocitrulline (nNOS inhibitor) inhibited nitrites (53.6 ± 6.8%; *n* = 8; *P* < 0.01 in HT). To test whether nitrite release was mediated by nNOS (a Ca^2+^/calmodulin dependent enzyme), nitrites were measured in the absence of Ca^2+^. Ca^2+^-free media decreased the nitrite contents in NT (Δ: 881.6 ± 37.2 pmol/mg; *n* = 7; *P* < 0.001) and in HT (Δ: 252.4 ± 43.1 pmol/mg; *n* = 6; *P* < 0.01). In any case, aminoguanidine did not modify the nitrite content in NT (Δ: 54.2 ± 77.4 pmol/mg; *n* = 7; *P*: NS) and in HT (Δ: 4.1 ± 53.8 pmol/mg; *n* = 10; *P*: NS). 

In HT, an increase of nitrites was observed with DPI (1024.7 ± 45.4 pmol/mg; *n* = 7; *P* < 0.001) and tempol (1071.2 ± 77.8 pmol/mg; *n* = 16; *P* < 0.001). In NT, these agents did not modify the nitrite contents. 

**Figure 3:**
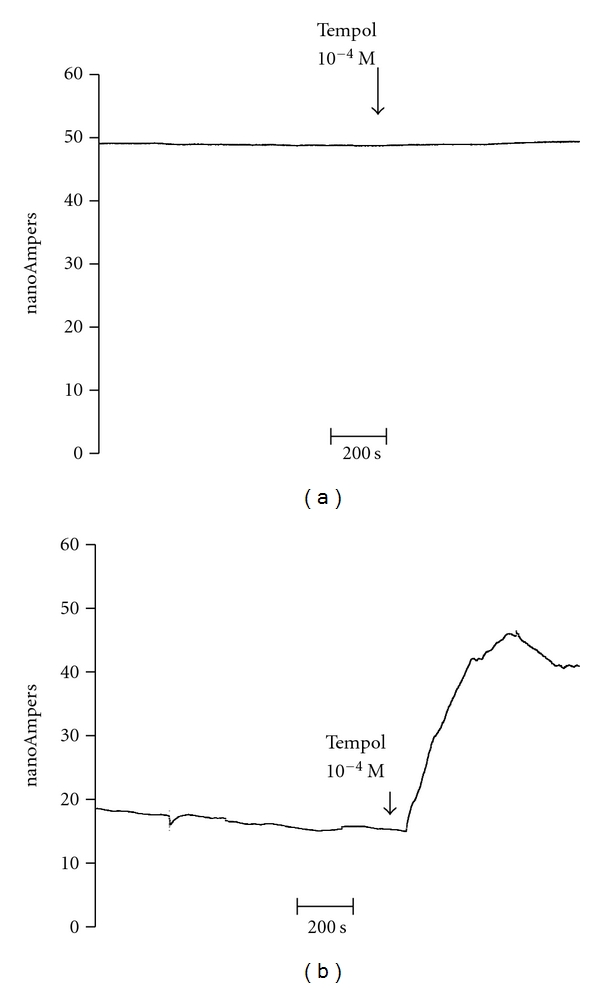
Typical experiment of direct measurement of NO in SV rings with endothelial dysfunction of one NT (upper) and one HT patient (lower) and the effect of tempol 10^−4^ M (arrows).

Figure 3 shows typical recorders of direct measurements of NO in basal conditions and the effect of tempol in NT (a) and HT (b). NT showed higher NO values than HT (48.8 ± 4.7 nA; *n* = 6 versus 17.2 ± 0.8; *n* = 7; *P* < 0.001). In HT, tempol 10^−4^ M increased NO release (42.5 ± 2.7 nA; *n* = 7; *P* < 0.001). In NT, tempol did not increase the NO contents (49.6±3.6 nA; *n* = 6; *P*: NS). Only in HT, DPI increased NO release (Δ: 24.5 ± 1.8 nA; *n* = 7; *P* < 0.001).

### 3.3. Presence of nNOS and eNOS by Immunohistochemistry

**Figure 4:**
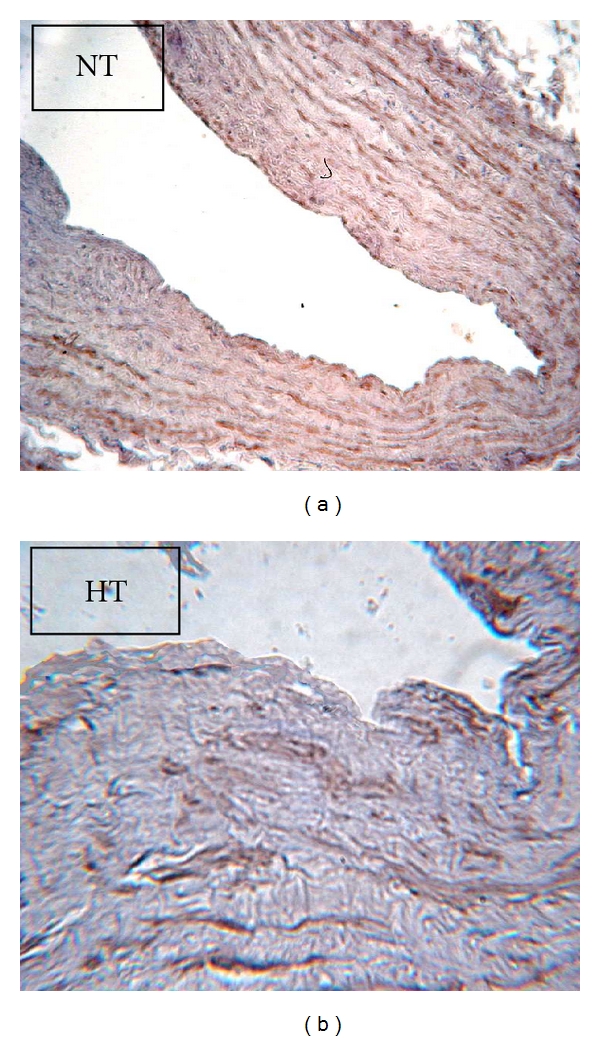
Microphotography (40x) of neuronal nitric oxide synthase (nNOS) immunohistochemistry of a transverse section of SV rings from one NT (a) and one HT patient (b). The ABC peroxidase method was used to generate the brown stain at the sites of primary antibody binding.

Because NO was present in SV rings without endothelium and based on previous findings from our laboratory in which we demonstrated the nNOS presence in VSMC of IMA rings [9], immunohistochemistry for nNOS was performed in SV. In both NT and HT, the anti-nNOS antibody specifically stained VSMC (Figure 4). The anti-nNOS-stained area was higher in NT than HT (26 ± 3.2% of the wall area; *n* = 7 versus 10 ± 0.6; *n* = 7, resp.; *P* < 0.001). No immunoreactive product was observed when samples were prepared without the primary antibody. 

In both NT and HT no staining with the anti-eNOS antibody was observed in the wall of SV rings.

### 3.4. Determination of Oxidative State

The levels of protein carbonyl groups were higher in HT than in NT (21.7 ± 4.4 pmol/mg protein; *n* = 5 versus 3.9 ± 0.8; *n* = 4; *P* < 0.05). 

Determinations of CD showed higher values in HT than in NT (0.26 ± 0.012 *μ*L/mg phosphates; *n* = 6 versus 0.17 ± 0.01; *n* = 6; *P* < 0.05).

The levels of GSSG were higher in HT than in NT (40.3 ± 16.3 *μ*mol/mg protein; *n* = 7 versus 4.5 ± 1.8; *n* = 7; *P* < 0.05). In addition, HT had elevated GSSG/GSH compared to NT (21.9 ± 8.6; *n* = 6 versus 3.1 ± 1.4; *n* = 7; *P* < 0.05).

Finally, TBARS levels were higher in HT than in NT (2.2 ± 0.6 nmol/mg protein; *n* = 6 versus 0.1 ± 0.01; *n* = 8; *P* < 0.01).

## 4. Discussion

We studied the contractility response to exogenous agonists in SV rings with endothelial dysfunction and the role of the oxidative stress on this response. Few studies have evaluated the vascular reactivity, or the impact of arterial hypertension, in SV. The SV is the vessel most commonly used for CABGS. However, the patency of this graft is controversial. Lytle et al. [28] have indicated that after 5 years of CABGS, the graft patency of SV exceeded that of IMA. Souza et al. [29] have suggested that SV provides high graft patency. However, Dashwood has indicated that the patency rate is poor, with a high proportion of patients requiring further surgery [30]. The mechanisms involved in the patency of SV are unclear. Some factors implicated are aging [31], serum cholesterol, diabetes, harvesting technique, or endothelial integrity. Ca^++^-protein kinase C-pathways are implicated in the reactivity of SV grafts [32]. In the present work, we found a hyperreactivity to both Ang II and NE in SV from HT patients, which was reverted by antioxidant agents. In this sense, hyperreactivity to vasoconstrictors has previously been observed in DOCA-salt hypertensive rats. Xu et al. [33] have observed increased reactivity to NE in mesenteric veins, and Li et al. [14] have reported a hyperreactivity to endothelin-1 in the vena cava, which was related to the presence of oxidative stress. In human SV, hypertension stimulates endothelin-1 release, which not only affects vascular reactivity but also increases oxidative stress [30].

In SV, Sharif et al. have studied the role of oxidative stress, indicating that N-acetylcysteine does not improve endothelial-dependent relaxation and VSMC function [34]. However, the same authors found that vitamin C improves the endothelial-dependent relaxation [17]. In our vessels, antioxidant agents diminish vascular reactivity, suggesting that oxidative stress is implicated in the hyperreactivity of HT. 

We also found that the antioxidant agents DPI and tempol increased NO release in SV. However, this NO is present in rings with endothelial dysfunction. This observation is in concordance with previous works from our laboratory on IMA [9, 10], in which we proposed that extraendothelial NO release is present through nNOS. Here, the extraendothelial NO presence in SV is supported by the observations that L-NAME and S-methyl-L-thiocitrulline decrease nitrite levels and that nNOS is present in VSMC (Figure 4). Another finding that support that the source of extraendothelial NO is through nNOS is the lack of staining with anti-eNOS antibody. In addition, the nNOS presence in SV has previously been demonstrated by Webb et al. [35].

Extraendothelial NO release was higher in NT than in HT. This result is in agreement with previous work in IMA [9]. To the best of our knowledge, no comparison of extraendothelial NO release in SV between HT and NT has been performed. DPI increased the NO contents in HT, suggesting a higher impact of the oxidative stress in HT patients. Few studies have been performed examining the role of oxidative stress in veins. O_2_^−^ production has been demonstrated to be higher in veins than in arteries [36, 37]. In human SV rings, Marlière et al. have observed that 5-series F2-isoprostanes (compounds produced by the nonenzymatic peroxidation of arachidonic acid) do not have vasomotor effects [38], and Antoniades et al. have shown that folic acid has beneficial effects on O_2_^−^ production [19]. 

O_2_^−^ has been demonstrated not only to be a NO-scavenger and a vasoconstrictor but also to produce direct vascular damage. In this sense, Shi et al. [36] have observed that biological changes in SV grafts are characterized by oxidative stress resulting from higher O_2_^−^ production and lower superoxide dimutase activity. In our work, the increased oxidative damage in hypertension is supported by the observation that the levels of protein carbonyl groups, CD, GSSG, GSSG/GSH; and TBARS are increased in HT rings. 

The present work demonstrates that extraendothelial NO counter-regulates contractility in SV used for CABGS. However, this NO action could be altered in hypertensive situations, even if there were no other associated risk factors. We suggest two mechanisms (1) increased oxidative stress and (2) a decreased ability of nNOS to produce NO. Further studies should be performed to evaluate the implications of these results in SV graft patency rates.

## Figures and Tables

**Table 1:** Clinical profile of the 44 study patients.

	HT (*n* = 30)	NT (*n* = 14)
Age, years	64 ± 2	61 ± 4
Sex, male/female	22/8	13/1
Body mass index	26.7 ± 0.6	25.4 ± 0.9
Systolic/diastolic blood pressure at the time of the hospitalization (mmHg)	122.9 ± 2/78.9 ± 2	116.7 ± 4/73.6 ± 4
Exsmokers, *n* (%)	14 (47)	9 (64)
Antecedents of dyslipidemia, *n* (%)	17 (57)	5 (36)

**Table 2:** Maximal contractile response (*R*_max⁡_) to Ang II and NE in SV rings.

	Ang II *R*_max⁡_ (mg)		NE *R*_max⁡_ (mg)	
	NT	HT	NT	HT
Krebs	581 ± 169 (7)^+++^	1193 ± 302 (7)	349 ± 132 (6)^+++^	951 ± 76 (6)
DPI 10^−5^ M	520 ± 163 (6)	680 ± 93 (6)***	314 ± 76 (6)	434 ± 31 (6)***
Tempol 10^−4^ M	580 ± 97 (6)	1195 ± 289 (7)	332 ± 20 (7)	880 ± 37 (7)
L-NAME 10^−4^ M	1380 ± 215 (6)*	1195 ± 289 (7)	947 ± 39 (6)**	955 ± 55 (7)

*P < 0.05 versus Krebs; **P < 0.01 versus Krebs, ***P < 0.001 versus Krebs; ^+++^ *P* < 0.001 HT versus NT. The numbers of experiments are given in parenthesis.
